# Photolithographic patterned surface forms size-controlled lipid vesicles

**DOI:** 10.1063/1.5002604

**Published:** 2018-01-02

**Authors:** M. Gertrude Gutierrez, Shotaro Yoshida, Noah Malmstadt, Shoji Takeuchi

**Affiliations:** 1 Institute of Industrial Science, The University of Tokyo, 4-6-1 Komaba, Meguro-ku, Tokyo, Japan; 2 Mork Family Department of Chemical Engineering and Materials Science, University of Southern California, 925 Bloom Walk, Los Angeles, California 90089, USA; 3 Mork Family Department of Biomedical Engineering, and Department of Chemistry, University of Southern California, 925 Bloom Walk, Los Angeles, California 90089, USA

## Abstract

Using traditional 2-D photolithographic methods, surface patterns are made on agarose and used to form lipid vesicles with controlled size and layout. Depending on the size and layout of the patterned structures, the lipid bilayer vesicle size can be tuned and placement can be predetermined. Vesicles formed on 2-D patterned surfaces can be harvested for further investigations or can be assayed directly on the patterned surface. Lipid vesicles on the patterned surface are assayed for unilamellarity and protein incorporation, and vesicles are indeed unilamellar as observed from outer leaflet fluorescence quenching. Vesicles successfully incorporate the integral membrane protein α-hemolysin and maintain its membrane transport function.

Giant unilamellar vesicles (GUVs) are biomimetic models of the cell plasma membrane; they are micrometer-sized vesicles made of a lipid bilayer.^1^ As plasma membrane models, they are heavily used to investigate the biophysics of lipids, integral membrane protein function, transport across lipid bilayers, and membrane mechanical properties.^2–6^ Current methods of GUV formation do not allow for precise control of the size or polydispersity of the GUVs formed.^7^ In methods that use hydrogels and dextran polymers for GUV formation, the pore size of a polymeric or hydrogel network can affect vesicle size, but vesical size distribution ranges can vary.^8^ Microfluidic methods can achieve monodispersity, but the possible contamination of organic solvents in microfluidic systems can be detrimental to lipid membranes.^8,9^ Other traditional methods of GUV formation, such as electroformation, gentle hydration, and the agarose rehydration method, cannot control for vesicle size distribution.^7,10–12^ Surface patterning techniques are an efficient platform for membrane formation with two methods, photolithography and microcontact printing, for forming size-controlled micrometer vesicles.^13–16^ In 2014, Wilkop *et al.* presented a patterned trehalose sacrificial layer for the formation of supported proteolipid-membranes.^13^ Using a microcontact printing approach, Howse *et al.* reported on a surface-patterned method for the formation of polymersomes, with unknown lamellarity.^14^ Furthermore, in both reports by Kang *et al.* in 2013 and by Taylor *et al.* in 2003, surface patterned techniques are performed to form size controlled vesicles that must rely on the electroformation method of GUV formation.^15,16^ While these approaches may offer size control of vesicles, they are limited to using specific bilayer formation techniques such as electroformation and gentle hydration which cannot concretely control vesicle placement and unilamellarity. Initial lipid films needed for electroformation or gentle hydration may not be single layer and may result in multilamellar GUVs.^15,16^ Because of these limitations, it is necessary to develop size-controlled easily assayable vesicle formation techniques using the agarose swelling method which can incorporate proteins, control placement, and form unilamellar vesicles. The agarose swelling method is an efficient approach to forming GUVs which does not rely on external power, such as a sonicator or alternating current.^11^ Here, we utilize photolithographic surface patterning of SU-8 for the formation and specific placement of GUVs using the agarose swelling method. The GUV size and placement was dependent on the size and layout of the surface patterns; GUVs formed on these photolithographically patterned surfaces were easily assayed for protein incorporation and unilamellarity and can be investigated as formed on the patterned surface, as shown in the results here, or may be harvested and investigated as unattached vesicles.

Figure 1 outlines the method used in surface-pattern agarose-coated glass substrates for controlled vesicle formation. First, 2% w/v of low-melting temperature agarose was spin-coated onto glass substrates. Agarose was allowed to gel at room temperature. Next, following traditional photolithographic methods, SU-8 was spin-coated onto the agarose.^17^ SU-8 was masked with various circular patterns, with the diameter of the circles being 2 *μ*m, 5 *μ*m, 10 *μ*m, or 20 *μ*m. The masked substrate was UV-cured and developed, resulting in a surface-patterned substrate (see supplementary material). Size-controlled vesicle formation was accomplished according to the agarose rehydration method.^10,11^ An o-ring was attached on top of the patterned area for confinement, and a thin film of 1,2-dioleoyl-*sn*-glycero-3-phosphocholine (DOPC) labeled with 0.2 mol. % rhodamine-1,2-dipalmitoyl-*sn*-glycero-3-phosphoethanolamine (Rh-DPPE) was drop-cast onto the patterned substrate. Excess solvent was removed with a stream of nitrogen gas, and the entire system was rehydrated with 1× phosphate buffered saline (PBS). Vesicles were imaged using epifluorescence microscopy.

**FIG. 1.**
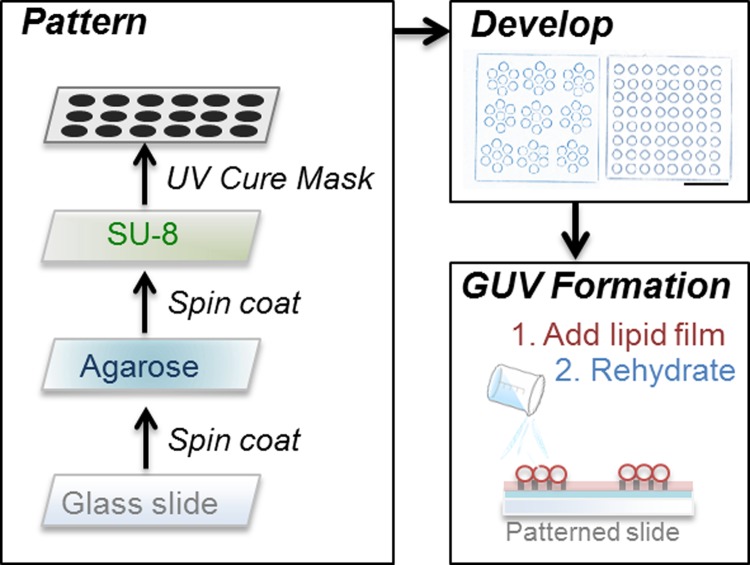
Fabrication of a patterned surface for size-controlled formation of GUVs. First, a clean glass slide is spin-coated with agarose and then spin-coated with SU-8. Then, a mask of the pattern is placed on top of the glass slide and UV-cured. The cured pattern is developed. An exemplary image of the pattern is shown with a scale bar of 50 *μ*m. For GUV formation, a lipid film is drop-cast onto the pattern and allowed to dry. The system is rehydrated with buffer, and vesicles of controlled radii are formed on the patterned surface.

Patterns were fabricated in gridded arrays and in flower arrays (circular geometries) (see Fig. 2). The pattern diameters that best resulted in monodisperse GUVs were 5 *μ*m and 10 *μ*m surface patterns as shown in Fig. 2. Monodisperse vesicles were successfully formed in the resist-free locations with the vesicle radius determined by the size of the pattern. 5 *μ*m patterned surfaces resulted in a mean vesicle radius of 6.8 ± 1.7 *μ*m, while 10 *μ*m patterned surfaces produced vesicles with a mean radius of 12.5 ± 3.3 *μ*m. As compared with GUVs grown on 2% agarose alone or on 2% agarose treated with SU-8 and developer without photomasking, the vesicle size distributions are better controlled and less polydisperse (supplementary material, Fig. S1). For the 2 *μ*m pattern, the high curvature stress of vesicles in that size range likely prevented overall formation, while for 20 *μ*m patterns, it is likely that there were multiple nucleation sites resulting in clusters of vesicles (supplementary material, Fig. S2).^18,19^ A key advantage of this approach is that GUVs formed on the patterned surface that are harvested and settled for observation and bulk assays maintain their size and shape. 5 *μ*m patterned GUVs harvested maintained a mean radius of 6.2 ± 17 *μ*m, and 10 *μ*m patterned GUVs harvested maintained a mean radius of 9.4 ± 3.6 *μ*m (supplementary material, Fig. S3). The slight differences in the radius, particularly for the 10 *μ*m pattern, could be due to the shear stress that the GUVs experience when pipetted and transferred.

**FIG. 2.**
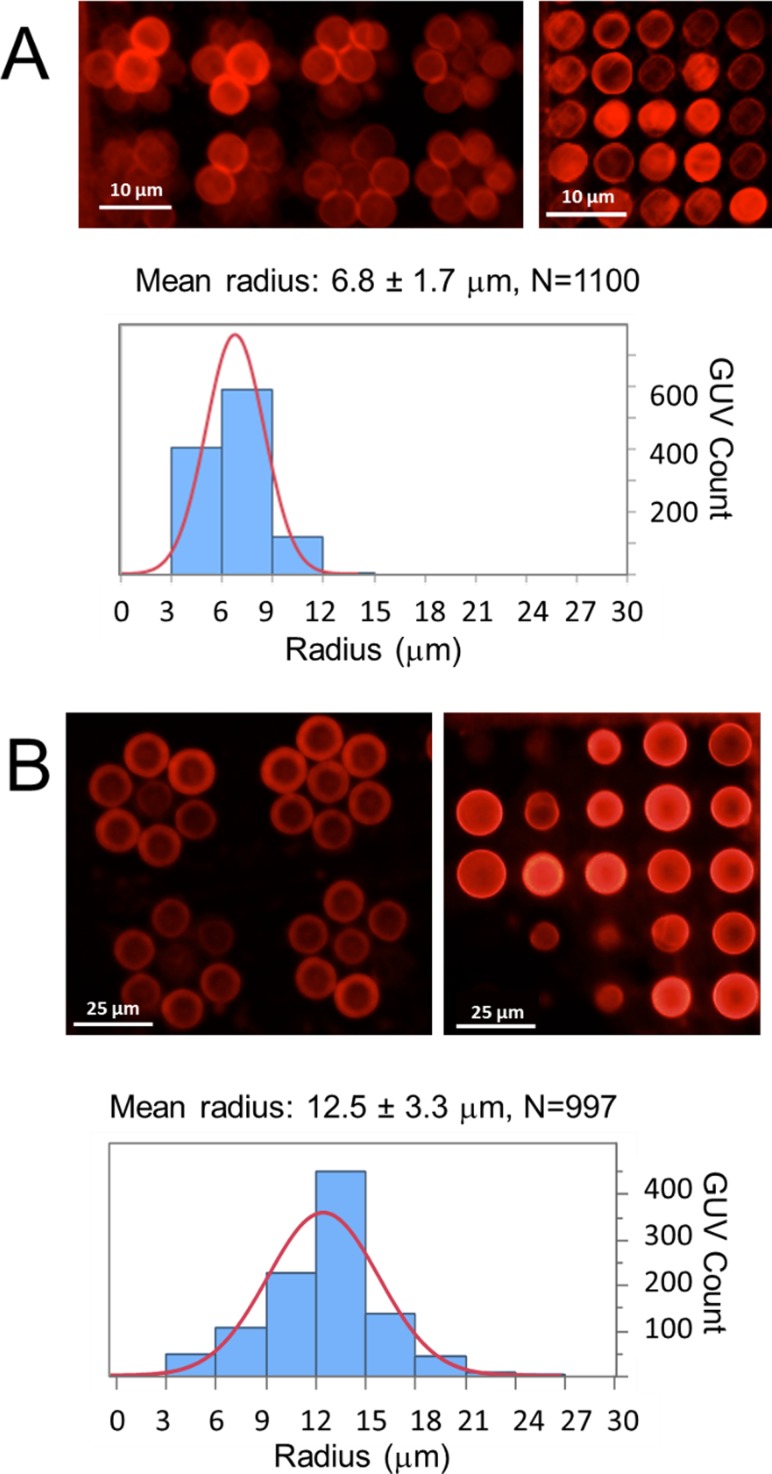
Controlled size of GUVs formed on patterned surfaces. Micrographs show fluorescence images of GUVs on SU-8 patterned agarose. (a) 5 *μ*m patterns show GUVs in flower and 5 × 5 arrays. GUVs on patterns have a mean radius of 6.8 ± 1.7 *μ*m as shown in the histogram of GUV size distribution. The scale bar is 10 *μ*m. (b) 10 *μ*m patterns in flower and 4 × 4 arrays. GUVs on 10 *μ*m patterns have a mean radius of 12.5 ± 3.3 *μ*m as shown in the histogram of GUV size distribution. The scale bar is 25 *μ*m. Errors are one standard deviation of the means. A normal distribution fit is shown in red over the histograms for ease of viewing.

Size controlled GUVs were formed on the photolithographically patterned surface and investigated for unilamellarity and the ability to probe the biophysics GUVs directly on the surface. In the first experiment, vesicles were quenched with QSY7, and in the second experiment, vesicles were incubated with α-hemolysin and transport across the bilayer membrane was observed (Fig. 3). In the first set of experiments, GUVs were formed on 10 *μ*m patterns and imaged. GUVs were then incubated in the dark for 10 min with a promiscuous contact quencher that does not cross the membrane, QSY7 (final concentration 0.1 mg/ml), and imaged to determine the amount of quenched fluorescence (see supplementary material for methods).^2,20^ After incubation, GUVs were imaged and intensity across entire micrographs before and after incubation was calculated. The micrographs analyzed contained roughly 200 GUVs and resulted in an intensity decrease of 45.5% ± 12.9% after quenching. The large standard deviation could be due to the background illumination from epifluorescence imaging. For visualization purposes in Fig. 3(a), the intensity across a single line is measured. ∼50% of the fluorescence intensity of the vesicles was quenched; since QSY7 can only access the outer leaflet of the bilayer, the fluorescence due to the inner leaflet remains. This indicates that the vesicles formed on the patterned surface are unilamellar.

**FIG. 3.**
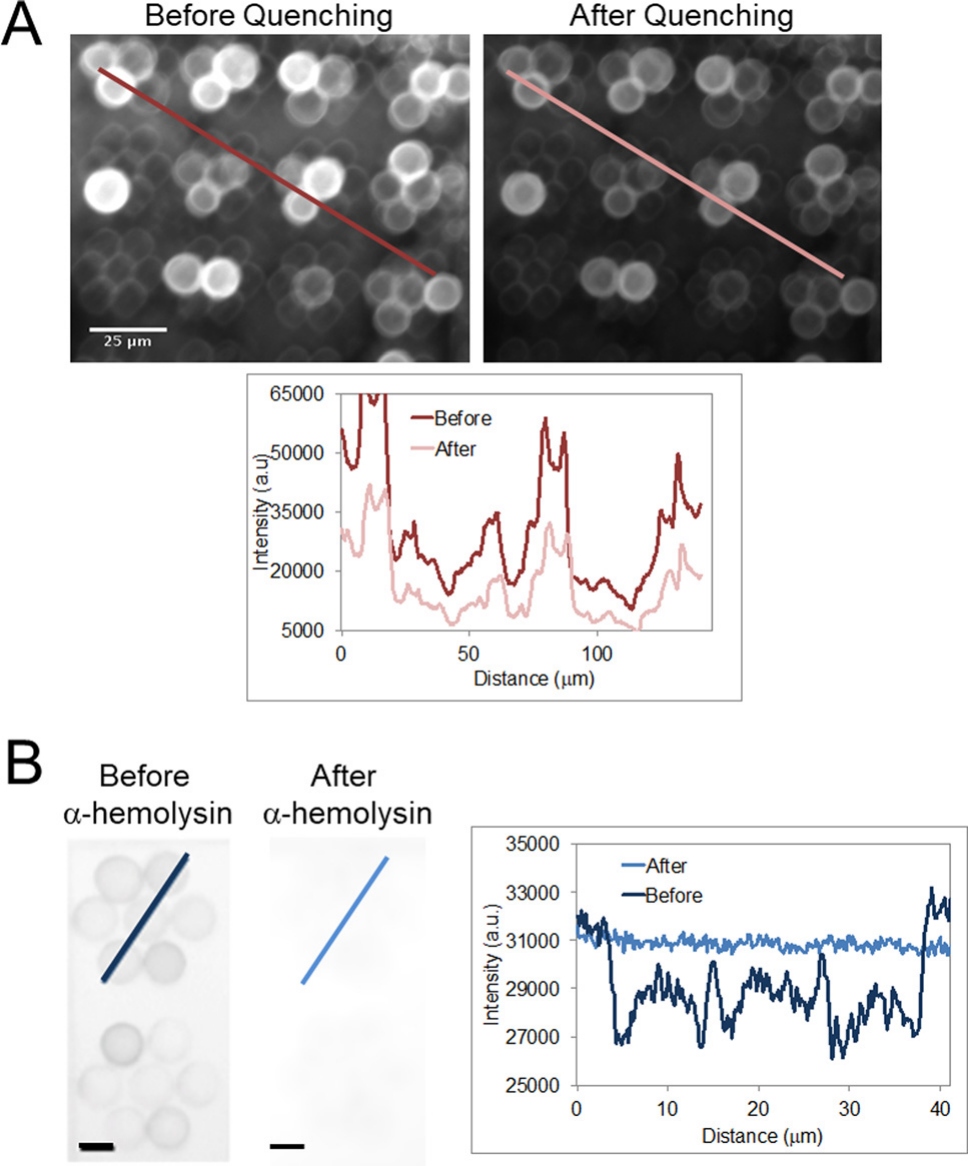
Assay of GUVs formed on patterned surfaces. (a) Micrographs of GUVs formed on the 10 *μ*m-patterned surface were imaged and incubated with QSY7 quencher for 10 min (before). After incubation, GUVs were imaged and intensity across entire micrographs before and after incubation was calculated. Across roughly 200 GUVs from these images, an intensity decrease of 45.5% ± 12.9% was observed. For visualization purposes, (a) shows intensity plots across indicated lines with ∼50% quenching of fluorescence across GUV bilayers. The scale bar is 25 *μ*m for both micrographs. (b) Micrographs of GUVs formed on the 10 *μ*m-patterned surface before and after incubation with α-hemolysin. After incubation with α-hemolysin, fluorescein from surrounding buffer is transported to the interior of vesicles as indicated by the increase in fluorescence intensity in the center of GUVs (also see supplementary material, Fig. S4). The scale bar is 10 *μ*m. Both sets of data indicate unilamellarity and ease of probing membrane properties of size controlled GUVs formed on the patterned surface.

Figure 3(b) further shows that this system produces physiologically relevant synthetic biomembranes by demonstrating that α-hemolysin, a protein known to immediately incorporate into the bilayer membrane and form a pore, can insert into the membranes of vesicles formed by this method.^21,22^ 10 *μ*m surface patterned GUVs were formed as described above. After formation, fluorescein, a fluorescent dye that does not passively cross the bilayer due to its charge at neutral pH, was added such that it surrounded the GUVs [final concentration 0.01 mg/ml, Fig. 3(b)].^21^ These vesicles were incubated with 100 nM (final concentration) α-hemolysin for 20 min and imaged before and after incubation. After incubation with α-hemolysin, fluorescein from the surrounding buffer is transported to the interior of vesicles as indicated by an increase in fluorescence intensity in the center of GUVs. This is shown in the after image in Figs. 3(b) and S4. Thus, the size-controlled GUVs formed on photolithographically patterned surfaces are unilamellar and can be easily used to investigate membrane proteins and transmembrane transport.

Photolithographic surface patterns on agarose allow for size-controlled vesicle formation with the average radius determined using the pattern size. Patterned GUVs can be placed according to pattern spacing and geometry. These GUVs are unilamellar and monodisperse, can be harvested for observations, and provide a platform to investigate membrane properties such as transport and protein incorporation on arrayed GUVs. The agarose method of GUV formation has successfully shown the incorporation of important proteins, including GPCRs, and the presented method now affords the ability to control the GUV size and placement for investigating membrane proteins in size-controlled lipid vesicles.^2,10,12,23,24^ Thus, this approach offers advantages and efficiencies for GUV fabrication as artificial cell models for probing membrane biophysics.

## METHODS

### Materials

DOPC (1,2-dioleoyl-*sn*-glycero-3-phosphocholine) and rhodamine-DPPE (1,2-dipalmitoyl-*sn*-glycero-3-phosphoethanolamine) were acquired from Avanti Polar Lipids. All reagents such as, but not limited to, low-melting-temperature agarose (Type I), phosphate buffered saline (PBS), chloroform (CHCl_3_), methanol (MeOH), quencher QSY7, fluorescein, isopropanol (IPA), acetone, sucrose, and glucose were obtained from Sigma Aldrich. Sykes-Moore chambers (Bellco), glass slides (thickness: 0.12–0.17 mm, width: 30 mm, and length: 40 mm, Matsunami Glass, Japan), and 18.2 MΩ cm Milli-Q water (EMD Millipore) were used in all experiments. SU-8 photoresist (SU8 2, Microchem) and SU-8 developer (Microchem) were used as per manufacturer's instructions.

### Fabrication of the patterned surface

Glass slides were cleaned by ultrasonication in an acetone bath for 10 min, washed with isopropanol, and dried with nitrogen gas. Then, the surface was made hydrophilic by oxygen plasma treatment. 300 *μ*l of 2% (w/v) agarose solution (Type I, Sigma, USA) at 50 °C was spin-coated on top of clean glass slides followed by 2 *μ*m-thick SU-8 photoresist (SU8 2, Microchem, USA) spin coating. The SU-8 layer was patterned by standard photolithography techniques; in brief, the SU-8 layer was exposed to a pattern of ultraviolet light through a photomask, developed by SU-8 developer (Microchem, USA), washed with isopropanol twice, and dried using nitrogen gas. Surface-patterned glass slides were kept under vacuum until use. The SU-8 thickness was determined to be 2 *μ*m using a profilometer. The SU-8 thickness was varied by using various volumes of SU-8 during spin-coating and was optimized such that the patterned surfaces were consistent and planar.

### Fabrication of giant unilamellar vesicles

Giant unilamellar vesicle formation was performed using methods previously described by Horger *et al.*, 2009, and Gutierrez *et al.*, 2014. Agarose-coated patterned surfaces as described above were used. A DOPC lipid film with 0.2 mol. % rhodamine-DPPE was made on the protein-agarose from a 3.3 mg/ml solution in CHCl_3_. A total of 10 *μ*l was used on a single cover slip, and lipid films were made by placing drops of lipid solution directly on top of patterned areas and avoiding unpatterned areas. In this work, the amount of lipid was not varied. Solvent was evaporated using a stream of nitrogen gas. An O-ring was placed on the patterned surface for liquid confinement, and the system was hydrated with 200 mM sucrose in PBS (pH 7.4). The films were swollen for 20 min in the dark. Bilayer vesicles were observed directly on the glass slides and were also harvested from the coverslip and settled in 3× of an isosmotic glucose solution (200 mM glucose in PBS, pH 7.4) and then transferred to observation chambers.

### Bilayer quenching experiment

GUVs were formed on 10 *μ*m photolithographic patterned surfaces as described above. QSY7 was added to GUVs formed on the patterned surface at a final concentration of 0.1 mg/ml and incubated in the dark for 15 min at room temperature. Microscopy images of the GUVs before and after QSY7 incubation were collected, and the intensity of entire micrographs was analyzed.

### Incorporation and transport across α-hemolysin

GUVs were formed on 10 *μ*m photolithographic patterned surfaces as described above. Upon formation, fluorescein was added to the solution at a final concentration of 0.01 mg/ml and allowed to equilibrate for 15 min. The system was then incubated with 100 nM (final concentration) of α-hemolysin for 20 min at room temperature. Microscopy images of the GUVs before and after incubation with α-hemolysin were collected.

### Microscopy

Imaging was done using an inverted microscope (IX71, Olympus, Japan) equipped with a CCD camera (DP72, Olympus, Japan) and a mercury lamp (USH-1030L, Olympus) or on an Axio Observer Z1 (Zeiss, Germany) inverted microscope using an EC Plan-Neofluar 20× objective and equipped with a Hamamatsu CMOS camera (Hamamatsu, Japan). Illumination for the Axio Observer Z1 was provided by a Colibri 2 LED Illumination System with a 120 V LED illuminator (Zeiss, Germany). Rhodamine-DPPE was illuminated using a red filter.

### Image processing and data analysis

All images were processed and analyzed using ImageJ. Particle analysis and measurements were performed using ImageJ Analyze Tools. All images are presented without any further processing adjustments or corrections and are scaled from minimum to maximum intensity. Statistical analysis was performed using JMP.

### Ethical statement

The authors declare that ethics approval is not required associated with this paper.

## SUPPLEMENTARY MATERIAL

See supplementary material for four supplementary figures (Fig. S1 to S4) and additional references.
